# Broccoli Biofumigation Reshapes the Rhizosphere Bacterial Community to Suppress *Fusarium oxysporum* and Reduce Potato Fusarium Wilt

**DOI:** 10.3390/jof12070478

**Published:** 2026-06-30

**Authors:** Dong Wang, Xiaofeng Su, Jiangyong Yu, Yuanzheng Zhao, Chao Zhang, Decai Jin, Hongyou Zhou, Ruibo Sun

**Affiliations:** 1 Key Laboratory of Biopesticide Creation and Resource Utilization in Inner Mongolia Autonomous Region, Inner Mongolia Agricultural University, Hohhot 010018, China; wangdong@imau.edu.cn (D.W.); yjy3427589701@163.com (J.Y.); 2 National Key Laboratory of Agricultural Microbiology, Biotechnology Research Institute, Chinese Academy of Agricultural Sciences, Beijing 100081, China; suxiaofeng@caas.cn; 3 Institute of Plant Protection, Inner Mongolia of Agricultural and Animal Husbandry Sciences, Hohhot 010018, China; zhaoyuanzheng.gege@163.com (Y.Z.); chaozhang0140@163.com (C.Z.); 4 Research Center for Eco-Environmental Sciences, Chinese Academy of Sciences, Beijing 100085, China; dcjin@rcees.ac.cn; 5 Anhui Province Key Lab of Farmland Ecological Conservation and Nutrient Utilization, College of Resources and Environment, Anhui Agricultural University, 130 Changjiangxilu, Hefei 230036, China; 6 Key Laboratory of JiangHuai Arable Land Resources Protection and Eco-restoration, Ministry of Natural Resources, 302 Fanhua Avenue, Hefei 230601, China

**Keywords:** soil-borne disease, microbial community assembly, *Fusarium oxysporum*, antagonistic bacteria

## Abstract

Biofumigation is increasingly recognized as an effective strategy for managing soilborne diseases. However, the understanding of the mechanisms of biofumigation has mostly focused on its direct inhibitory effects on plant pathogens, while the rhizosphere microbe-mediated effects induced by biofumigation remain unclear. Here, we investigated the effects of broccoli (*Brassica oleracea* var. *italica*) biofumigation on potato Fusarium wilt caused by *Fusarium oxysporum* and elucidated the changes in rhizosphere bacterial assemblage under biofumigation. Results showed that biofumigation significantly reduced disease incidence and increased tuber yield. In vitro assays revealed a strong direct inhibition of *F. oxysporum* by broccoli biofumigation, but the inhibition rate decreased from 99.78% on the first day to 76.27% on the seventh day. High-throughput sequencing and culture-based analyses demonstrated that biofumigation significantly shifted bacterial community assemblage in potato rhizosphere, enriching antagonistic taxa against *F. oxysporum*. Functional prediction suggested that biofumigation enriched bacteria associated with nitrogen consumption and methylotrophy. The changes in the rhizosphere bacterial community showed significant correlations with the incidence and severity of Fusarium wilt, indicating that biofumigation indirectly enhanced crop resistance to plant pathogens by altering the rhizosphere microbial community. These findings extend the current understanding of biofumigation beyond direct chemical toxicity and classical antibiosis and highlight its potential as an ecological strategy that harnesses the plant-associated microbiome for disease management.

## 1. Introduction

Potato (*Solanum tuberosum* L.) is the most important non-cereal food crop, which is cultivated on over 17.2 million hectares worldwide, with annual production exceeding 370 million tons [1]. As a staple food for more than one billion people, potato provides essential carbohydrates, vitamins, and minerals, contributing significantly to food security and rural livelihoods, particularly in lower-income countries [2]. However, Fusarium wilt, one of the most destructive diseases caused by the soilborne fungus, such as *Fusarium oxysporum*, adversely affects potato production worldwide [3]. The pathogen produces resilient resting structures, spores, that can survive in soil for more than a decade without a susceptible host [4]. After germinating, spores infect roots, travel through epidermal cells or wounds, and colonize the vascular tissue, where hyphae develop and block water transport, causing wilting [5,6,7]. The long-term persistence of pathogenic fungi in soil, combined with a broad host range and the lack of highly resistant potato cultivars, makes Fusarium wilt particularly difficult to manage [8].

Conventional control strategies, including crop rotation, chemical fumigation, and soil solarization, have shown limited efficacy or face increasing regulatory and environmental constraints [8]. Crop rotation can reduce plant diseases, but its effectiveness is often limited by the specific adaptation of rotation crops to local conditions. Moreover, rotation crops may inadvertently promote the proliferation of other pathogens, thereby complicating disease management in those regions [9,10]. Chemical fumigants such as chloropicrin and metam sodium can reduce soil inoculum levels but are broad-spectrum biocides that may disrupt beneficial soil microbiota and raise environmental concerns [11,12]. Consequently, there is growing interest in developing ecologically sound alternatives that suppress soilborne pathogens while preserving or enhancing soil microbial health [13,14].

Biofumigation, defined as the incorporation of glucosinolate-containing plant tissues into soil, has emerged as a promising non-chemical approach for managing soilborne diseases. Upon hydrolysis by endogenous myrosinase, glucosinolates release isothiocyanates (ITCs), which are volatile compounds with broad antifungal and nematicidal activities [15]. Previous studies have demonstrated that biofumigation can reduce the incidence of Fusarium wilt in types of crops [8,16,17]. This effect has traditionally been attributed to the direct fungitoxicity of volatile compounds and secondary plant metabolites (e.g., isothiocyanates and glucosinolates) against pathogens [16,18]. Broccoli (*Brassica oleracea* var. *italica*) is a particularly excellent biofumigant crop due to its high glucosinolate content, quick biomass production, and suitability for temperate cropping systems [19,20]. However, the effect of biofumigation on reducing potato Fusarium wilt is still unknown.

In addition, accumulating evidence suggests that the suppressive effects of biofumigation are not solely explained by chemical-mediated pathogen inactivation. Incorporation of fresh organic matter into soil also triggers profound shifts in the soil microbiome, including the enrichment of specific bacterial taxa with antagonistic properties [21,22,23]. For instance, biofumigation with brassica residues has been shown to stimulate populations of *Bacillus*, *Pseudomonas*, *Streptomyces*, and other genera known to produce antifungal metabolites, compete for nutrients, or induce systemic resistance in plants [23,24,25]. These microbially driven pathways may play a significant role in disease suppression, particularly after the fast volatilization and destruction of ITCs. Nonetheless, it is unclear how much biofumigation influences the structure and function of the rhizosphere bacterial population and whether such changes directly correlate to increased antagonistic activity against *F. oxysporum* and decreased Fusarium wilt incidence in potato.

The rhizosphere, a small area of soil affected by plant roots, is a habitat for a wide range of microorganisms. Plant health and disease resistance are directly related to the composition and functional features of this microbiome [26,27,28]. Beneficial microorganisms in the rhizosphere can suppress soilborne pathogens through multiple mechanisms, including the production of antimicrobial compounds, competition for nutrients and niches, and the induction of systemic resistance in the host plant [29,30]. Therefore, the rhizosphere microbiome acts as the “biological barrier” of defence against pathogen invasion, and harnessing rhizosphere microbial community assembly and functions is considered a potential approach to increase plant growth and health [31,32,33,34]. A study revealed that adding amino acids, such as L-isoleucine, L-ornithine, L-valine, L-serine, and β-alanine, could promote the germination and root colonization of beneficial bacteria and ultimately promote plant growth [35]. In addition, growing evidence indicates that agricultural strategies would also impact rhizosphere microbial assembly and functions through indirect pathways of regulating plant metabolisms and rhizosphere environments [36,37]. For example, our previous study has revealed that foliar humic acid application is a top-down regulatory approach to modify rice rhizosphere bacterial communities and enhance phosphorus mobilization [38]. Therefore, biofumigation may also affect the rhizosphere microbial community by altering the metabolic processes of the crop. However, the impact of biofumigation on rhizosphere microbial community assemblage and the associations between rhizosphere microbial community and the occurrence of Fusarium wilt under biofumigation are still unknown.

In the present study, the effects of broccoli biofumigation on the occurrence of potato Fusarium wilt and on the rhizosphere bacterial community assemblage and functional traits in three different soils were detected. We hypothesize that biofumigation would shape an antagonistic bacterial community against *F. oxysporum* in the potato rhizosphere, thereby helping to reduce the incidence of Fusarium wilt. The findings would provide new insights into the microbiome-mediated mechanisms of biofumigation and support the development of microbial-based strategies for sustainable management of Fusarium wilt in potato.

## 2. Materials and Methods

### 2.1. Pot Experiment

Naturally infested soils containing pathogens of potato Fusarium wilt were collected from three sites in Inner Mongolia: Hohhot (IM), Ulanqab (JN), and Wuchuan (WC). Soils were air-dried, homogenized, and sieved (3 mm) before use (Shangyu Wusi Test Instrument Factory, Shaoxing, China). Before potting, subsamples of the sieved soils from the three locations were collected to determine their baseline physicochemical properties (texture, organic matter content, total N, available P and K, and electrical conductivity). Soil samples collected from three locations were subjected to biofumigation treatment (BF), and untreated samples served as control (Control). Each treatment contained five replicates. Filter paper (qualitative medium speed; Hangzhou Xinhua Paper Industry Co., Ltd., Hangzhou, China) was placed at the bottom of each pot (upper inner diameter 15 cm, lower diameter 10 cm, height 10 cm; Taizhou Hongda Plastic Products Co., Ltd., Taizhou, Zhejiang, China). To standardize the amount of soil across pots, the water content of each fresh soil was determined by oven-drying a small subsample (50 g) at 105 °C for 48 h (DHG-9140A; Shanghai Yiheng Scientific Instrument Co., Ltd., Shanghai, China). Based on this measurement, the fresh weight of each soil needed to provide 1000 g dry weight equivalent was calculated and used to fill each pot. The BF treatment was added with 10 g of broccoli leaves as follows: Fresh broccoli (*Brassica oleracea* var. *italica*) leaves collected at the curd stage were chopped into 1 cm segments and thoroughly mixed with the soil. All the pots were sealed with plastic film for 3 months. Then two virus-free seed potatoes (*Solanum tuberosum* variety “Favorita”; Inner Mongolia Academy of Agricultural and Animal Husbandry Sciences, Hohhot, China) were planted per pot. Standard horticultural management was maintained throughout the growth period.

### 2.2. Determination of Disease Incidence and Disease Index

Plant growth parameters including height, stem diameter, and fresh/dry biomass were measured at 100 days post-planting (dpp). Disease severity was evaluated at 100 dpp based on the scoring criteria summarized in Table 1.

Disease incidence and disease index were calculated using the following formulas:

$$\mathrm{Disease}\;\mathrm{incidence}\;(\%)\;=\frac{\mathrm{Number}\;\mathrm{of}\;\mathrm{diseased}\;\mathrm{plants}}{\mathrm{Total}\;\mathrm{number}\;\mathrm{of}\;\mathrm{investigated}\;\mathrm{plants}}\times 100$$

$$\mathrm{Disease}\;\mathrm{index}=\frac{\sum \mathrm{Number}\;\mathrm{of}\;\mathrm{plants}\;\mathrm{at}\;a\;\mathrm{specific}\;\mathrm{disease}\;\mathrm{grade}\times \mathrm{Corresponding}\;\mathrm{grade}\;\mathrm{value}}{\mathrm{Total}\;\mathrm{number}\;\mathrm{of}\;\mathrm{investigated}\;\mathrm{plants}\times \mathrm{Highest}\;\mathrm{grade}\;\mathrm{value}}\times 100$$

### 2.3. Measurement of Effect of Biofumigation on F. oxysporum Growth

The pathogenic *Fusarium oxysporum* strains used in this study were isolated directly from symptomatic, wilted potato plants in the pot experiments. Diseased root tissues were surface-sterilized, rinsed, and incubated on potato dex-trose agar (PDA) plates. Subsequent morphological observation and molecular identification (via PCR amplification and sequencing of the ITS region) confirmed that the pathogenic isolates recovered from all three distinct soil treatments belonged to the identical *F. oxysporum* strain. To definitively prove its pathogenicity, a bioassay was conducted following Koch’s postulates. Healthy potato seedlings were inoculated with a conidial suspension of the isolated strain. After typical wilt symptoms developed, which were identical to those observed in the original pot experiments, the exact same fungal strain was successfully reisolated from the infected plants. This conclusively confirmed the isolated strain as the primary causal agent of the observed potato wilt disease.

The inhibitory effect of broccoli residues on the mycelial growth of fungal pathogens was determined using the growth rate method. About 9 mm diameter mycelial plugs were placed in the middle of freshly prepared potato dextrose agar (PDA) plates. To create the fumigation environment, 12 g of surface-sterilized by hydrogen peroxide, crushed broccoli residue was placed into the bottom half of a separate, sterile Petri dish. The PDA plate containing the fungal plug was then inverted and placed directly over the base containing the broccoli residue, effectively replacing its lid. The two dishes were tightly sealed together with Parafilm to create a closed double-dish fumigation system. Plates without broccoli residues served as controls. Each treatment was performed with four replicates and incubated in the dark at 25 °C. Colony diameters were measured daily for 7 days using the cross-intersection method. To accurately reflect net mycelial growth, the initial plug diameter was factored into the denominator. After algebraically simplifying the numerator (where the initial plug diameters cancel each other out), the final inhibition rate (%) was calculated as follows:

$$Inhibition\;rate\left(\%\right)=\frac{Dc-Dt}{Dc-Dp}\times 100$$

where *Dc* is the colony diameter of the control, *Dt* is the colony diameter of the treatment, and *Dp* is the diameter of the initial mycelial plug (9 mm).

### 2.4. Isolation of Antagonistic Strains Against F. oxysporum

Soil samples (1 g) from three sites were suspended in 100 mL sterile saline (0.85% NaCl) and serially diluted. Aliquots (100 µL) of appropriate dilutions (10^−4^ to 10^−6^) were spread onto LB agar, nutrient agar (NA), and King’s B medium. Plates were incubated at 28 °C for 48 h. Single colonies with distinct morphologies were purified by streaking onto fresh plates. A total of 120 bacterial isolates were obtained and stored in 20% glycerol at −80 °C. The antagonistic activity of bacterial isolates against *F. oxysporum* was evaluated using the dual-culture method on potato dextrose agar (PDA). A mycelial plug (5 mm diameter) taken from the margin of an actively growing *F. oxysporum* culture was placed at the centre of a PDA plate. Four bacterial isolates were streaked equidistantly (2 cm from the centre) around the fungal plug. Plates inoculated only with the fungal plug served as controls. All plates were incubated at 25 °C for 7 days. The isolated strains were identified by 16S rRNA gene sequencing using the universal primers 27F (5′-AGAGTTTGATCATGGCTCAG-3′) and 1492R (5′-TACGGTTACCTTGTTACGACTT-3′).

### 2.5. Soil Sampling, DNA Extraction, and Bacterial Community Analysis

At 100 dpp, potato plants were carefully uprooted from each pot. The loosely adhering soil was removed by gentle shaking, and the tuber-associated rhizosphere soil was carefully collected by brushing the roots and tubers with a sterile brush [39,40,41]. For each pot, the rhizosphere soil from the two plants was pooled to obtain one composite sample per pot. The samples were immediately flash-frozen in liquid nitrogen and stored at −80 °C for subsequent microbial analysis.

Soil total DNA was extracted from the rhizosphere soil using a standard commercial kit. The V1–V9 hypervariable region of the bacterial 16S rRNA gene was amplified using primer sets 27F/1492R. The purified amplicons were subjected to sequencing using the Pacbio SMRT platform at Biomarker Technologies Corporation (Beijing, China).

Bioinformatic analysis of the sequencing data was performed as described in previous studies [28,38,40,41,42,43,44,45]. The adapters and primer sequences were removed using Cutadapt (version 1.18) [43]. Then VSEARCH (version 2.30.4) was used to join the paired-end sequences, filter the low-quality and chimeric sequences, and generate zero-radius operational taxonomic units (zOTUs) [44]. Based on the SILVA database (version 138.1), taxonomic assignment of zOTUs was performed using the “SINTAX” function (sintax_cutoff = 0.9) [45]. The non-target zOTUs (not assigned as bacteria) were removed for further analysis. Functional traits of the bacterial community were predicted using FAPROTAX (version 1.2.12).

The 16S rRNA gene sequences of the isolated antagonistic strains were aligned to the representative sequences of ZOTUs obtained from high-throughput sequencing of rhizosphere bacterial communities. Matches were assigned based on 100% sequence similarity. Three strains C1, C29, and BS-7 were assigned as ZOTU1874, ZOTU464, and ZOTU985, respectively.

### 2.6. Field Experiment

Field experiment was conducted in the Inner Mongolia Jining District of Ulanqab City (41°2′3.5″ N, 113°6′40.0″ E) to evaluate the effects of broccoli biofumigation on potato Fusarium wilt in practice. There were two treatments: biofumigation (BF) and control. Each treatment contained five replicate plots. The plot size was 27 m^2^ (2.7 m × 10 m). The application dosage of the BF treatment was determined with reference to published studies [46] with fresh broccoli residuals added at 15,000 kg/ha, equal to 200 g per planting hole. The residues were fully incorporated into the topsoil in the planting zone during the May 2024 sowing season. The experiment used a randomized design, with five replicate plots per treatment. In October 2024, at harvest stage, tuber yield and disease severity were assessed using the grading criteria specified in Table 1. The incidence and disease index of Fusarium wilt were calculated accordingly.

### 2.7. Statistical Analysis

Statistical analyses were performed in the R platform (version 4.5.0) according to the methods described in previous studies [41,47,48]. The significance of differences among treatments was checked by the Kruskal–Wallis rank-sum test (*p* < 0.05) using the “dplyr” library. The analysis referring to bacterial community, including calculation of Chao1 richness, non-metric multidimensional scaling (NMDS) based on Bray–Curtis distances, and the distribution of zOTUs among treatments was performed using the “microceo” library. ANOSIM (Analysis of Similarities) was performed using the “anosim” function in the R package ‘vegan’ (999 permutations) to test for significant differences among treatment groups. βNTI (β-nearest taxon index) was calculated to show changes in deterministic and stochastic processes during soil microbial community assembly using the “picante” library. The figures were drawn using the “ggplot2” library.

## 3. Results

### 3.1. Effects of Biofumigation on Potato Growth and Fusarium Wilt Incidence

**Figure 1:**
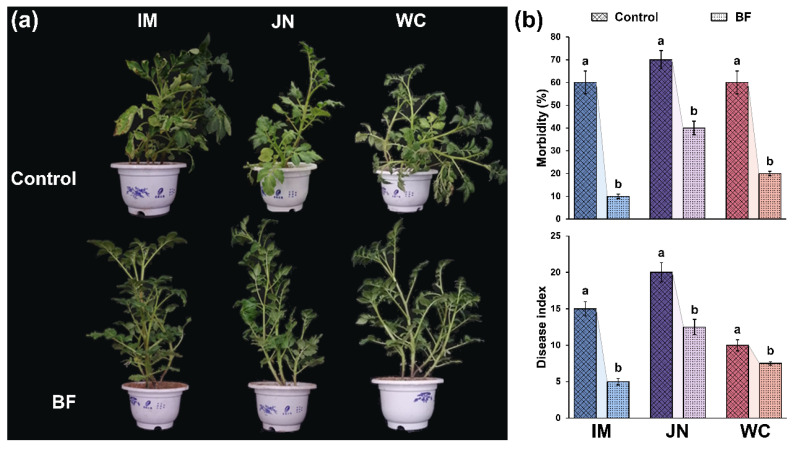
(**a**) The phenotypes of potato plants under different treatments: (**b**) The impact of BF treatment on the incidence and disease index of potato Fusarium wilt. Different letters indicate a significant difference between the Control and BF treatment within the same soil (*p* < 0.05). Control: treatment without biofumigation; BF: treatment with biofumigation. IM: soil from Hohhot; JN: soil from Ulanqab; and WC: soil from Wuchuan.

In the three soils with different physicochemical properties (Table 2), biofumigation showed consistently positive effects on potato growth (Figure 1a). The biofumigation treatment significantly increased plant height, plant fresh and weight, shoot fresh weight, and root fresh weight. However, it showed little impact on root length in the soil from JN and WC, while it significantly improved root length in the soil from IM (Table 3).

Biofumigation decreased the incidence and degree of Fusarium wilt across the three soils (Figure 1b). And the effect was greatest in the soil from IM, in which the incidence of Fusarium wilt decreased from 60% to 10%, while biofumigation resulted in 30% and 40% decreased in Fusarium wilt incidence in JN and WC soil. Biofumigation also reduced the severity of Fusarium wilt on potato, as represented by significant decrease in disease index.

### 3.2. Effects of Biofumigation on F. oxysporum Growth

**Figure 2:**
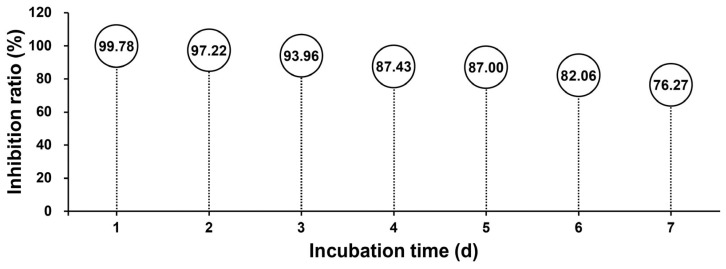
The effect of biofumigation on *F. oxysporum* growth. The bubble plot shows the inhibition ratio during the incubation.

Through a plate assay experiment, the effect of biofumigation on *F. oxysporum* growth was determined. Results showed that biofumigation showed a great antagonistic effect on *F. oxysporum* growth. During the 7 days of incubation, the size of *F. oxysporum* colony in the control plate gradually increased. On the seventh day, the colony had almost completely covered the entire plate. On the plates of the BF treatment, the colony size of *F. oxysporum* barely increased during the first three days; although it gradually increased from the fourth day, the colony size remained far smaller than that in the control plate. The inhibition ratio of biofumigation against *F. oxysporium* gradually decreased with prolonged incubation (Figure 2).

### 3.3. Effects of Biofumigation on Bacterial Community in Potato Rhizosphere

**Figure 3:**
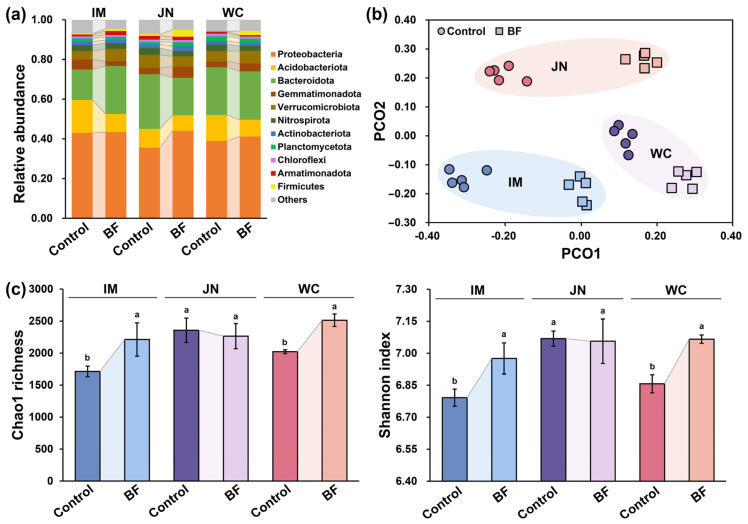
(**a**) Bacterial community composition in rhizosphere of potato under different treatments. (**b**) PCoA plot showing the variation in rhizosphere bacterial community in different treatments. (**c**) The impact of BF treatment on bacterial diversity in potato rhizosphere. Different letters above the bars indicate significant difference between Control and BF treatments with the same soil (*p* < 0.05). Control: treatment without biofumigation; BF: treatment with biofumigation. IM: soil from Hohhot; JN: soil from Ulanqab; and WC: soil from Wuchuan.

The results of high-throughput sequencing showed that Proteobacteria, Acidobacteriota, and Bacteroidota were the three dominant phyla (relative abundance > 10%) in the potato rhizosphere (Figure 3a). The impact of biofumigation on rhizosphere bacterial taxonomic composition varied across different soils. For example, biofumigation decreased the dominance of Acidobacteriota in IM and WC soils, while it had little impact on JM soil. However, there is a consistent phenomenon that biofumigation significantly increased the relative abundance of Firmicutes in all three soils.

The PCoA plot clearly showed the difference in rhizosphere bacterial community among treatments (Figure 3b). Soils from different sites were clearly separated from each other, indicating baseline site-specific community variation. What is more, within the soils from the same site, the soils under BF treatment were separated from the control soils, showing that BF treatment shaped a distinct bacterial community. This was further confirmed by the results of ANOSIM (Table S1), which showed that bacterial communities of the three control soils were significantly different from each other (*p* < 0.05) and the bacterial communities of the BF treatments were significantly different from their corresponding control (*p* < 0.05).

Chao1 richness and the Shannon index were calculated to infer the α diversity of the bacterial community. Results showed that BF treatment significantly increased bacterial Chao1 richness in soils from WC and NC, but had little impact on soil from JN (Figure 3c). The same patterns were also observed for the Shannon index.

### 3.4. Effects of Biofumigation on the Functional Profiles of Bacterial Community

**Figure 4:**
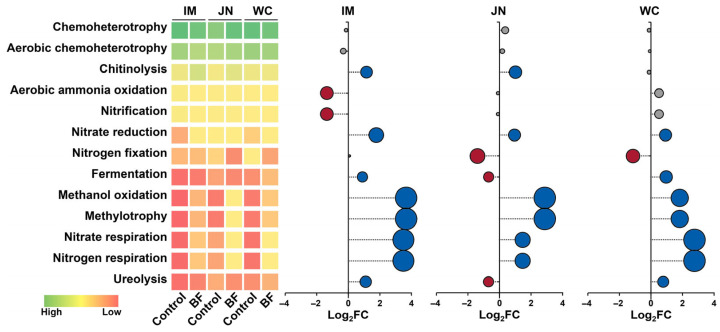
The impact of BF treatment on bacterial functional profiles predicted by FAPROTAX. The red bubbles indicate significantly lower BF treatment compared with Control, while the blue bubbles indicate significantly higher BF treatment compared with Control.

Through FAPROTAX, the functional profiles of the bacterial community were predicted. Results showed that the effects of biofumigation on bacterial functional profiles varied across soil types (Figure 4). Chemoheterotrophy and aerobic chemoheterotrophy were the two domain auxotypes, and they were little impacted by BF treatment. There were several specific changes in bacterial functions to BF treatments in specific soils. Aerobic ammonia oxidation and nitrate reduction showed a negative response to BF treatment in the IM soil, while ureolysis function was negatively impacted by BF treatment in the JN soil. However, there was a consistent pattern that BF treatment significantly increased the relative abundance of functions involved in nitrate reduction, methanol oxidation, methylotrophy, nitrate respiration, and nitrogen respiration.

### 3.5. Effects of Biofumigation on the Antagonistic Bacteria Against F. oxysporum

**Figure 5:**
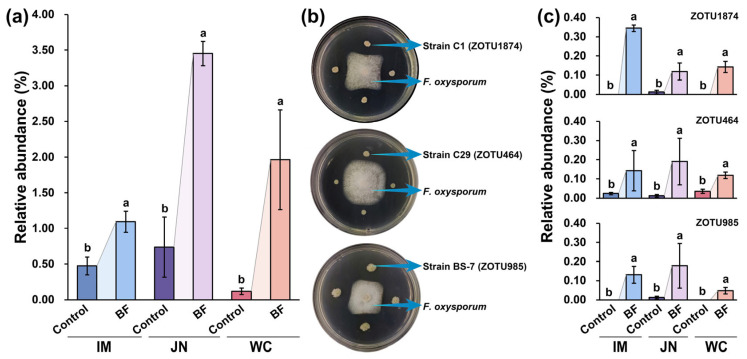
(**a**) The relative abundance of genus *Bacillus* in soils under different treatments. (**b**) The antagonistic effects of strain C1, C29, and BS-7 against *F. oxysporum*. (**c**) The relative abundance of the antagonistic bacteria in soil under different treatments. Different letters indicate significant difference between Control and BF treatments within the same soil. Control: treatment without biofumigation; BF: treatment with biofumigation. IM: soil from Hohhot; JN: soil from Ulanqab; and WC: soil from Wuchuan.

Many *Bacillus* strains have been demonstrated to possess antagonistic activity against *F. oxysporum*. In the present study, the relative abundance of genus *Bacillus* was significantly increased by BF treatment, and this result was observed in all three soils (Figure 5a). Several antagonistic bacterial strains were also isolated in the present study, and three of them (strains C1, C29, and BS-7) were also detected in the result of high-throughput sequencing, which were assigned as zOTU1874, zOTU464, and zOTU985, respectively. They showed a clear antagonistic effect on *F. oxysporum* in the plate (Figure 5b). The relative abundance of these three antagonistic strains in the whole bacterial community was significantly higher in the BF treatment than in the control (Figure 5c), and the same patterns occurred in all three detected soils.

### 3.6. Effects of Biofumigation on Bacterial Community Assembly

**Figure 6:**
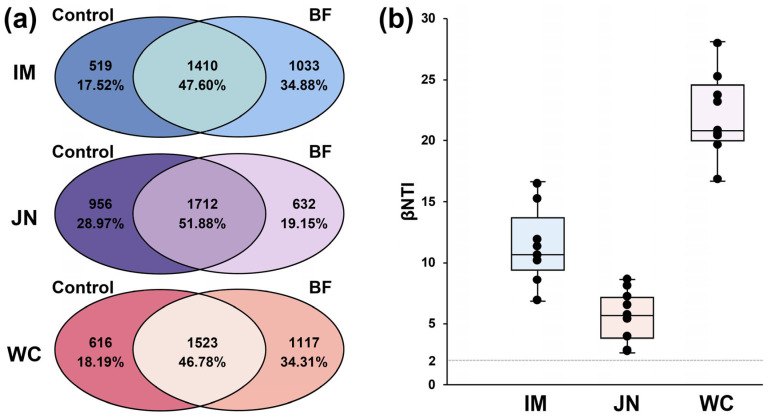
The impact of BF treatment on bacterial zOTU distribution (**a**) and community assembly (**b**). Control: treatment without biofumigation; BF: treatment with biofumigation. IM: soil from Hohhot; JN: soil from Ulanqab; and WC: soil from Wuchuan.

In all three studied soils, most of the zOTUs were shared in both the control and BF soils (Figure 6a), showing most of the zOTUs were retained in the BF-amended soils (Figure 6a). However, 17.82% to 28.97% zOTUs were not detected in the BF soils, showing that BF treatment acted as a filter of soil bacteria, while 19.15% to 34.88% zOTUs were unique in the BF-amended soils, indicating colonization of new taxa in the BF-amended soils.

βNTI values were calculated to explore the impact of BF treatment on rhizosphere bacterial community assembly (Figure 6b). In all three studied soils, the βNTI values between control and BF treatments were all larger than 2, indicating that the variation in bacterial community between treatments was dominated by deterministic processes. However, the βNTI values between soils varied greatly. The largest βNTI values were observed in the soil from WC, followed by the soil from IM and JN. The effects of biofumigation treatment on bacterial community assembly differ among different soils.

### 3.7. Correlations Between Bacterial Community and Potato Incidence

Results from Mantel tests showed that the incidence and disease index of potato Fusarium wilt were significantly correlated with bacterial community (Table 4). In addition, they also had significant correlations with the relative abundance of the potential antagonistic bacteria (ZOTU1874, ZOTU464, and ZOTU985) and some functional profiles of the rhizosphere bacterial community, including methanol oxidation, methylotrophy, nitrate reduction, nitrate respiration, and nitrogen respiration (Table 4).

### 3.8. Effects of Biofumigation on Potato Health and Yield in Field Experiment

**Figure 7:**
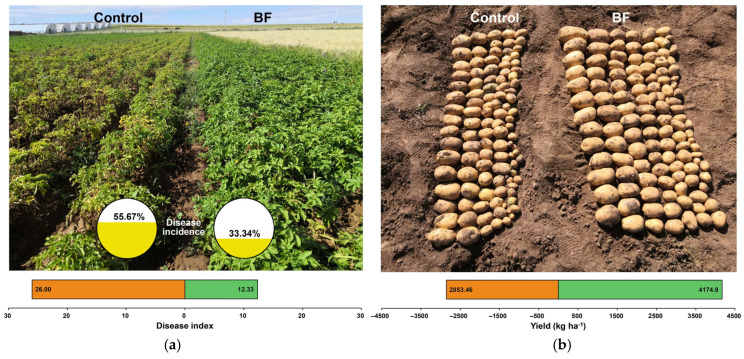
(**a**) Incidence and disease index of potato Fusarium wilt in the field experiment. (**b**) Potato yield under different treatments. Control: treatment without biofumigation; BF: treatment with biofumigation.

In the field experiment, biofumigation treatment showed a significant effect on reducing Fusarium wilt in potato and increasing potato yield (Figure 7). Compared with the control treatment, BF treatment significantly decreased the incidence of Fusarium wilt from 55.67% to 33.34% and decreased the disease index by 53%. Potato yield in the Control treatment was 2853.46 kg ha^−1^, while BF treatment significantly increased potato yield by 46.31%, reaching 4174.9 kg ha^−1^.

## 4. Discussion

Biofumigation is an effective agricultural practice to decrease plant disease. The results from the present study further confirmed that broccoli biofumigation significantly reduced the incidence of potato Fusarium wilt in different soils and increased potato yield in practice. We further revealed that broccoli biofumigation shaped a bacterial community enriched with antagonistic taxa against *F. oxysporum* in the potato rhizosphere. This finding moves beyond the traditional view that biofumigation acts solely through direct chemical toxicity and instead highlights a possible role of microbiome-mediated suppression.

The traditional paradigm of biofumigation emphasizes the direct, chemical-based suppression of soilborne pathogens through the release of bioactive glucosinolate hydrolysis products, particularly isothiocyanates [15,49]. Our results provide clear evidence that broccoli biofumigation exerts a strong direct inhibitory effect on *F. oxysporum* growth (Figure 2). This direct inhibition of pathogens is consistent with a previous report that Brassica biofumigation effectively inhibited pathogen growth, which was related to the release of ITCs from Brassica tissues. An effector may disrupt fungal cell membranes, inactivate key enzymes, and reduce spore germination and mycelial development [50,51]. The fact that inhibition was most pronounced during the early phase (≥90% within three days) suggests a rapid release of ITCs upon incorporation of fresh broccoli residues, followed by a gradual decline in inhibitory activity as ITCs volatilize [52]. Nevertheless, the persistent effect (>75% at day 7) indicates that residual ITCs continue to suppress the pathogen, but the efficiency was much lower than the previous day, indicating that the direct inhibitory effect of broccoli biofumigation on the pathogen gradually decreases over time. Therefore, the direct effect alone does not fully explain the sustained protection throughout the growing season (Figure 1), because ITCs are volatile and relatively short-lived in soil [53]. Consequently, while direct chemical inhibition of *F. oxysporum* is a crucial initial step in biofumigation-mediated disease suppression, additional indirect mechanisms—such as nutrient input and the recruitment of antagonistic bacterial communities in the rhizosphere—likely contribute to long-term protection.

In addition to the direct chemical effects of isothiocyanates, the high quantity of broccoli residues applied in our field experiment introduced a large pulse of organic matter rich in carbohydrates, proteins, and fibre. This nutrient input likely stimulated the growth of copiotrophic microorganisms, thereby increasing their dominance in the microbial community. Consequently, this would enhance the competition of these species against plant pathogens, thereby inhibiting the growth of the pathogens. A previous study has demonstrated that organic matter incorporation significantly changed soil microbial communities and diluted plant pathogens in soil [54,55,56]. At the same time, the input of organic materials, such as straw and manure, would also increase plant growth and resistance to biotic and abiotic stresses, increasing the resistance of plant to pathogen infection and modifying rhizosphere microbial communities [57,58,59,60]. The present results further demonstrate that broccoli biofumigation fundamentally altered the assembly processes of the rhizosphere bacterial community. Specifically, biofumigation drove community assembly toward deterministic selection (Figure 6). This change is ecologically significant due to the fact that deterministic assembly, which is encouraged by environmental filtering and biotic interactions, results in communities that are more functionally specialized and predictable [61,62]. This phenomenon suggested that biofumigation strengthened the regulation of plants on the rhizosphere bacterial community assembly. As a result, the deterministic selection imposed by biofumigation consistently enriched bacterial taxa with known antagonistic activities against *F. oxysporum*, including members of *Bacillus* (Figure 5), and such taxa showed significantly negative correlations with Fusarium wilt occurrence (Table 4). Once enriched, these antagonistic bacteria may suppress *F. oxysporum* through multiple mechanisms, including antibiosis, competition for iron and other nutrients, and induction of systemic resistance in potato plants [63]. Importantly, deterministic selection often strengthens species interactions and niche differentiation, potentially increasing the functional redundancy and stability of the beneficial community [64]. In the soils with low bacterial diversity (IM and WC), biofumigation treatment significantly increased rhizosphere bacterial diversity (Figure 3c), which implies an increase in the stability and functional diversity of the rhizosphere bacterial community. This finding provides a mechanistic explanation for the longevity of disease suppression beyond the short-lived direct chemical effects of isothiocyanates. While the volatile isothiocyanates dissipate, the establishment of an antagonistic rhizosphere microbiome that can function as a “biological barrier” against the pathogen continues to protect the host.

Our findings show that biofumigation of broccoli significantly changes the ecological roles of the microbial community in the rhizosphere, in addition to directly enriching hostile bacteria. In particular, we found that the methylotrophic bacteria and microorganisms involved in nitrate and nitrogen respiration were much more abundant in the potato rhizosphere after biofumigation (Figure 4). This functional shift suggests that biofumigation creates a competitive environment where key growth-limiting resources—particularly nitrogen and one-carbon compounds (e.g., methanol)—are rapidly cycled and consumed by the activated resident microbiota [65,66]. Competition for essential nutrients is a fundamental mechanism of biological control in the rhizosphere [63]. *F. oxysporum*, like many soilborne fungal pathogens, depends on the availability of simple nitrogen sources (e.g., ammonium, nitrate, amino acids) and carbon substrates for germination, hyphal growth, and host infection [67,68]. The enrichment of nitrogen-consuming microorganisms can accelerate the transformation of inorganic nitrogen into organic forms or gaseous losses, thereby reducing the pool of readily available nitrogen that the pathogen could exploit. Methylotrophic bacteria use one-carbon molecules, which *F. oxysporum* may employ as an alternate carbon source or energy source when it is saprophytic in soil [69]. It is plausible that by competing with the pathogen for these specific substrates, the enriched functional guilds may limit *F. oxysporum* growth and activity in the rhizosphere, potentially reducing its inoculum potential and subsequent infection success.

Nutrient competition functions constantly and is independent of the synthesis of diffusible antibiotics or volatile substrates, making it a viable, long-term suppression mechanism. In contrast to direct chemical suppression, which declines rapidly post-biofumigation, functional alterations in microbial communities may last for weeks or even months, provided that the modified resource availability and biotic interactions are sustained [70]. This persistence aligns with our observation that the direct inhibitory effect against *F. oxysporum* decreased greatly after 7 days (Figure 2), but the disease incidence and severity of potato in the biofumigation treatment were significantly reduced throughout the entire growth period, and the yield was significantly increased (Figure 7). Moreover, the simultaneous enrichment of both nitrogen-consuming and methylotrophic bacteria indicates that multiple resource competition pathways may act in concert, creating a “nutrient-depleted barrier” around potato roots that impedes pathogen establishment [71].

Although the effects of biofumigation on bacterial community and its potential associations with Fusarium wilt have been illustrated, one limitation of this study is that we did not characterise the fungal community responses to biofumigation. Fungi, including potential antagonists such as *Trichoderma* and the target pathogen *Fusarium*, may also be influenced by biofumigation and could contribute to disease suppression or pathogen load dynamics [72]. Future studies should integrate both bacterial and fungal community analyses to provide a more holistic view of the microbiome-mediated effects of biofumigation. In addition, the magnitude of plant improvement and disease reduction by biofumigation did not simply correlate with the baseline disease incidence in control soils. For example, JM and WC soil had a similar incidence, but biofumigation resulted in a reduction of 50 percentage points of incidence in JM soil, whereas only a reduction of 40 percentage points in WC soil (Figure 1b). This lack of a simple linear relationship suggests that soil characteristics—such as soil physicochemical properties (e.g., organic matter content), the initial pathogen load, indigenous microbial community composition, or the specific competitiveness of resident antagonists—may modulate the efficacy of plant disease control [15,73,74]. Future studies should systematically investigate how soil properties and baseline microbiome characteristics influence the responsiveness of disease suppression to biofumigation.

## 5. Conclusions

The present study demonstrated that broccoli biofumigation consistently reduces the incidence and severity of potato Fusarium wilt caused by *F. oxysporum* across different soils while simultaneously increasing tuber yield under field conditions, confirming the broad-spectrum efficacy of this approach. Critically, we revealed that broccoli biofumigation increased the diversity and reshaped the rhizosphere bacterial community, enriching bacterial taxa with antagonistic activity against *F. oxysporum*. Moreover, functional prediction showed that biofumigation altered the ecological functions of the rhizosphere microbiome, notably by enriching nitrogen-consuming and methylotrophic bacteria. These changes helped to increase the competition of bacterial community with *F. oxysporum* and contributed to a sustained disease-suppressive effect. These findings revealed that biofumigation not only directly suppresses the pathogen but also reshapes the rhizosphere microbiome for long-term protection, which moves beyond the traditional view of biofumigation as solely a chemical soil treatment and highlights its potential as an ecologically sound strategy that harnesses the plant-associated microbiome for disease management.

## Figures and Tables

**Table 1:** Disease severity grading scales for potato Fusarium wilt.

Grade	Fusarium Wilt
0	No symptoms
1	<25% leaves wilted; slight vascular yellowing
2	25–50% leaves wilted; <50% vascular browning
3	50–75% leaves wilted; most vasculars browned
4	>75% leaves wilted; complete vascular browning

**Table 2:** Physicochemical properties of the soil used in the present study.

Region	Soil Organic Matter (%)	Available Nitrogen (mg/kg)	Available Potassium (mg/kg)	Available Phosphorus (mg/kg)
IM	2.10 ± 0.07	30.19 ± 1.68	36.64 ± 1.28	33.98 ± 2.60
WC	2.65 ± 0.11	33.25 ± 2.02	38.74 ± 1.77	27.97 ± 1.66
JN	2.18± 0.22	34.13 ± 1.62	40.95 ± 3.58	44.38 ± 0.90

**Table 3:** Effects of broccoli biofumigation treatment on potato growth profiles.

Soil	Treatment	Plant Height (cm)	Root Length (cm)	Plant Fresh Weight (g)	Plant Dry Weight (g)	Shoot Fresh Weight (g)	Root Fresh Weight (g)
IM	Control	35.58 ± 0.80 b	15.67 ± 0.90 b	12.72 ± 0.85 b	1.09 ± 0.19 b	11.39 ± 0.84 b	1.29 ± 0.05 b
	BF	40.91 ± 1.83 a	18.04 ± 1.22 a	18.62 ± 2.46 a	1.32 ± 0.15 a	17.05 ± 2.45 a	1.58 ± 0.05 a
JN	Control	30.33 ± 1.34 b	17.66 ± 0.66 a	13.86 ± 1.41 b	1.24 ± 0.12 b	11.83 ± 1.32 b	2.15 ± 0.16 b
	BF	33.00 ± 1.22 a	18.03 ± 1.17 a	17.73 ± 1.73 a	1.47 ± 0.07 a	12.77 ± 1.81 a	4.96 ± 1.47 a
WC	Control	36.76 ± 1.94 b	18.14 ± 0.73 a	14.79 ± 0.75 b	1.38 ± 0.10 b	13.52 ± 0.74 b	1.27 ± 0.14 b
	BF	39.78 ± 1.01 a	18.51 ± 0.75 a	18.34 ± 1.88 a	1.46 ± 0.06 a	16.30 ± 1.74 a	2.03 ± 0.29 a

Data is mean ± SD (*n* = 5). Data is collected at the 100th day. Different letters indicate a significant difference between the Control and BF treatment within the same soil (*p* < 0.05). Control: treatment without biofumigation; BF: treatment with biofumigation. IM: soil from Hohhot; JN: soil from Ulanqab; and WC: soil from Wuchuan.

**Table 4:** Spearman’s correlation coefficients between potato Fusarium wilt profiles and rhizosphere bacterial profiles.

	Incidence	Disease Index
Bacterial community	0.67	0.64
ZOTU1874	−0.76	−0.73
ZOTU464	−0.69	−0.70
ZOTU985	−0.77	−0.81
Methanol oxidation	−0.64	−0.70
Methylotrophy	−0.61	−0.62
Nitrate reduction	−0.59	−0.61
Nitrate respiration	−0.65	−0.58
Nitrogen respiration	−0.64	−0.67

Only the significant correlations (*p* < 0.05) were shown.

## Data Availability

The data presented in this study are available upon request from the corresponding authors.

## Supplementary Materials

The following supporting information can be downloaded at: https://www.mdpi.com/article/10.3390/jof12070478/s1, Figure S1: Morphological characteristics of the pathogenic *Fusarium oxysporum* isolate; Table S1: The results of ANOSIM.
