# Supplementary material for: Broccoli Biofumigation Reshapes the Rhizosphere Bacterial Community to Suppress Fusarium oxysporum and Reduce Potato Fusarium Wilt
Source: J Fungi (Basel). 2026 Jun 30;12(7):478. doi: 10.3390/jof12070478 (PMC13412287; doi:10.3390/jof12070478)
Supplement: Supplementary file 1 [file jof-12-00478-s001.zip › Supplementary File S1.pdf]

## 1. Morphological Observation of the Pathogenic Isolate

The purified fungal strain was incubated on potato dextrose agar (PDA) plates at 25 °C in darkness for 7 days for morphological characterization. The colony formed fluffy, cottony aerial mycelium that was white in the early growth stage, and gradually developed pale purple to dark reddish-purple pigmentation on both the front and reverse sides of the medium. Microscopic observation showed typical sickle-shaped macroconidia with 3–5 septa, as well as abundant oval microconidia borne on false heads, all of which matched the classical morphological features of *Fusarium oxysporum*.

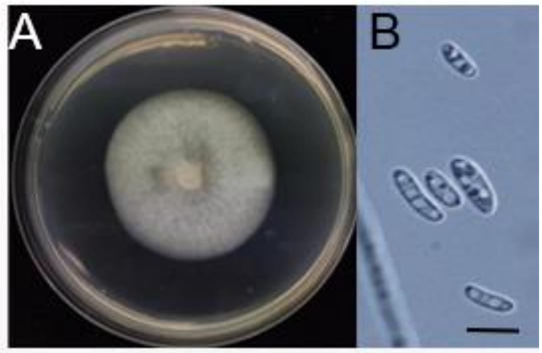


Figure S1 Morphological characteristics of the pathogenic *Fusarium oxysporum* isolate. (A) Front view of a 7-day-old colony on PDA medium; (B) Microscopic morphology of microconidia (scale bar = 10 μm).

## 2. Molecular Identification via ITS Region Sequencing

Genomic DNA of the purified isolate was extracted using the CTAB method. The internal transcribed spacer (ITS) region of fungal ribosomal DNA was amplified with the universal primer pair ITS1 (5′-TCCGTAGGTGAACCTGCGG-3′) and ITS4 (5′-TCCTCCGCTTATTGATATGC-3′). Purified PCR products were subjected to Sanger sequencing.

The obtained ITS sequence was aligned against the NCBI GenBank nucleotide database via BLASTn. The top alignment hits all corresponded to *Fusarium oxysporum* with ⩾ 99.8% sequence identity and 100% query coverage, confirming the species-level taxonomic identity of the isolate. The ITS sequences of isolates from all three soil treatments were 100% identical, verifying that they belonged to the same single strain.

Supplementary Sequence S1. Full-length ITS sequence of the pathogenic isolate (FASTA format):

> Fusarium oxysporum
ACATTACCGAGTTTACAACTCCCAAACCCCTGTGAACATACCACTTGTTGCCTCGGCGG
ATCAGCCCGCTCCCGGTAAAACGGGACGGCCCGCCAGAGGACCCCTAAACTCTGTTTC
TATATGTAACTTCTGAGTAAAACCATAAATAAATCAAAACTTTCAACAACGGATCTCTT
GGTTCTGGCATCGATGAAGAACGCAGCAAAATGCGATAAGTAATGTGAATTGCAGAAT
TCAGTGAATCATCGAATCTTTGAACGCACATTGCGCCCGCCAGTATTCTGGCGGGCATG
CCTGTTCGAGCGTCATTTCAACCCTCAAGCACAGCTTGGTGTTGGGACTCGCGTTAATT
CGCGTTCCCCAAATTGATTGGCGGTCACGTCGAGCTTCCATAGCGTAGTAGTAAAACCC
TCGTTACTGGTAATCGTCGCGGCCACGCCGTTAAACCCCAACTTCTGAATGTTGACCTC
GGATCAGGTAGGAATACCCGCTGAACTTAAGCATATCAAAA
