# Supplementary material for: Broccoli Biofumigation Reshapes the Rhizosphere Bacterial Community to Suppress Fusarium oxysporum and Reduce Potato Fusarium Wilt
Source: J Fungi (Basel). 2026 Jun 30;12(7):478. doi: 10.3390/jof12070478 (PMC13412287; doi:10.3390/jof12070478)
Supplement: Supplementary file 1 [file jof-12-00478-s001.zip › Table S1.pdf]

Table S1 The results of ANOSIM

Comparison	R	*P*
IM control vs JN control	0.436	0.001
IM control vs WC control	0.397	0.001
JN control vs WC control	0.421	0.002
IM control vs IM BF	0.501	0.009
JN control vs JN BF	0.519	0.013
WC control vs WC BF	0.443	0.001

Control: treatment without biofumigation; BF: treatment with biofumigation. IM: soil from Hohhot; JN: soil from Ulanqab; WC: soil from Wuchuan.
